# Humeral Diaphyseal Osteolytic Lesion as the Initial Presentation of Acute Myeloid Leukemia in a Child: A Case Report and Review of the Literature

**DOI:** 10.7759/cureus.22791

**Published:** 2022-03-03

**Authors:** Atsushi Goto, Takahiro Iwata, Satoshi Nozawa, Haruhiko Akiyama

**Affiliations:** 1 Department of Orthopaedic Surgery, Gifu University Graduate School of Medicine, Gifu, JPN

**Keywords:** iliac crest bone marrow biopsy, bone marrow examination, magnetic resonance imaging, skeletal symptoms in children, acute myeloid leukemia

## Abstract

It is well known that acute myeloid leukemia (AML) is characterized by lethargy, fever, pallor, and purpura. In children, however, skeletal symptoms may be present at onset in rare cases, and such cases tend to be misdiagnosed as osteomyelitis or septic arthritis. To distinguish acute leukemia from osteomyelitis or bone tumor, the utility of magnetic resonance imaging (MRI) has been discussed. We present a pediatric case of AML in which the initial manifestation was pain in a single bone, and the diagnosis was aided by bone marrow examination and MRI. A one-year-old male with AML presented with left humeral bone pain and intermittent fever. T1-weighted magnetic resonance imaging (T1WI) revealed diffuse low signal intensity in the bone marrow adjacent to the localized musculoskeletal symptoms. Despite a lack of blasts in the peripheral blood, the histopathological features of the bone focus suggested the need for an iliac crest bone marrow biopsy to obtain a definitive diagnosis. After the diagnosis of AML, the patient received induction and consolidation chemotherapy. He is currently alive in remission after a post-diagnosis follow-up of 36 months. To date, only seven pediatric cases of AML with skeletal symptoms at initial presentation have been reported, including the present one. In three cases, the skeletal lesion was observed at a single site, and the initial misdiagnosis was discitis, septic arthritis, or acute osteomyelitis. We suggest that AML should be considered as a differential diagnosis in children presenting with treatment-resistant single skeletal lesions. Not only MRI but also bone biopsy can yield diagnostically important information.

## Introduction

The presenting signs of acute myeloid leukemia (AML) are usually lethargy, fever, pallor, and purpura [1]. Most patients with AML are diagnosed by iliac crest bone marrow biopsy after the appearance of blasts in the peripheral blood. However, skeletal symptoms as an initial presenting sign have not been documented. Here, we report a one-year-old male with AML in whom the presenting sign was a humeral osteolytic lesion, which led to an initial misdiagnosis of acute osteomyelitis. T1-weighted magnetic resonance imaging (T1WI) depicted diffuse low signal intensity of the bone marrow in a region adjacent to localized bone pain. Histopathological examination of the bone focus proved diagnostically informative. The patient received induction and consolidation chemotherapy, resulting in the disappearance of the blasts. We suggest that AML should be considered as a differential diagnosis for children presenting with single bone lesions.

## Case presentation

**Figure 1:**
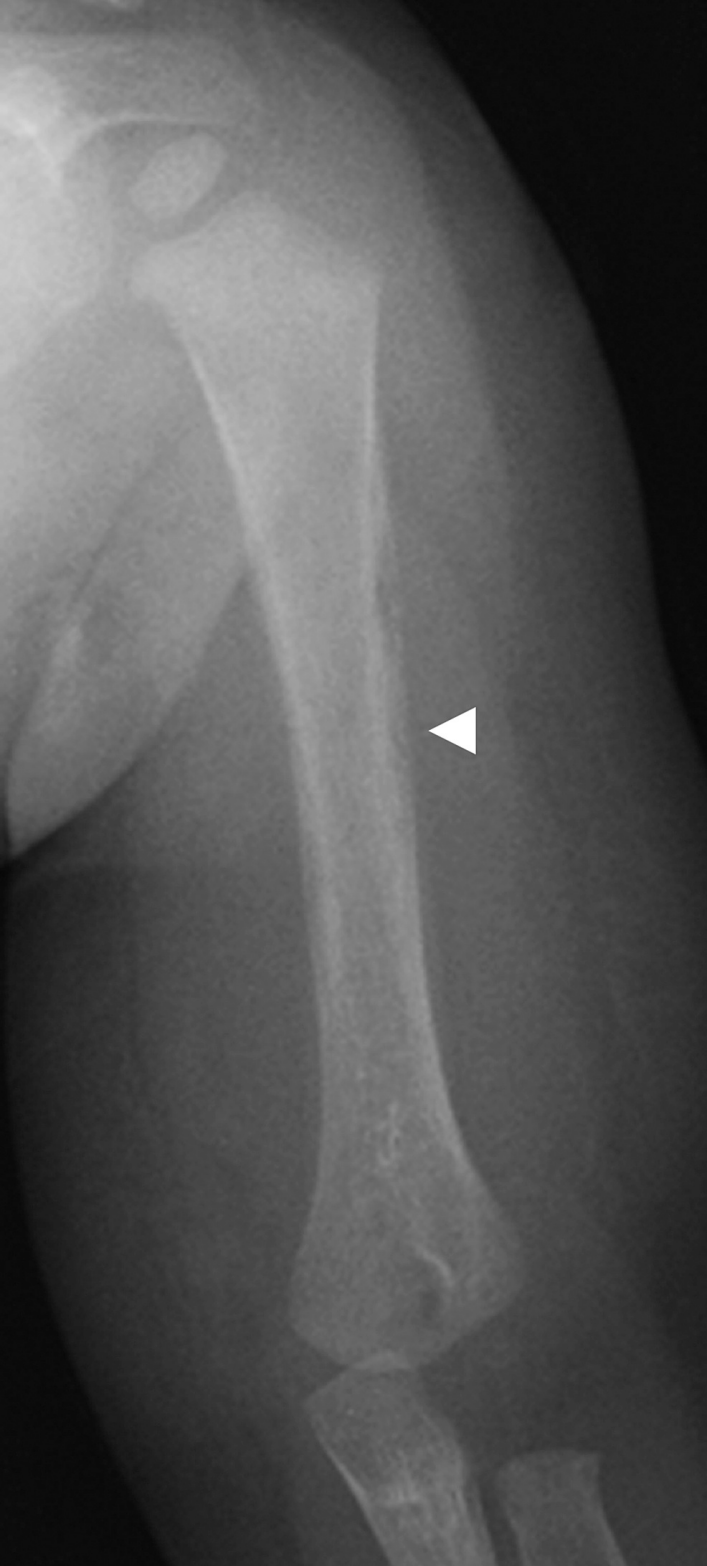
Anteroposterior radiograph of the left humerus taken at the first visit showing a diaphyseal osteolytic bone lesion and a periosteal reaction (arrowhead).

**Figure 2:**
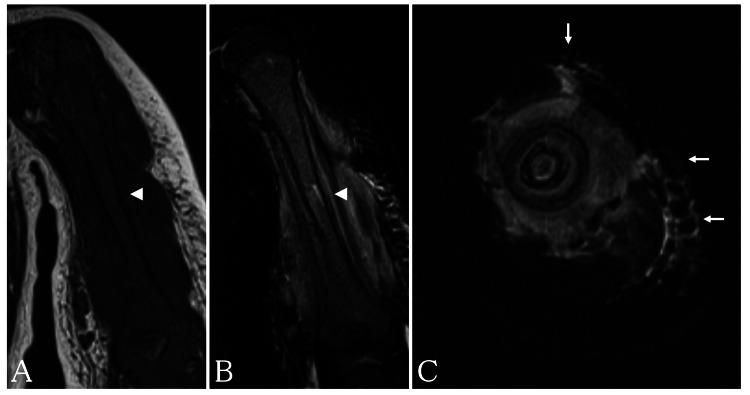
Magnetic resonance image of the left humerus. Coronal T1-weighted image shows diffuse low signal intensity in the left humeral bone marrow (arrowhead) (A), and a coronal short tau inversion recovery (STIR) image depicts local high signal intensity in the left humeral bone marrow (arrowhead) (B). Axial STIR image reveals high signal intensity in the subcutaneous tissues adjacent to the brachial muscles (arrows) (C).

A one-year-old male was referred to our department complaining of pain in the upper arm and intermittent fever. There was swelling, local heat, and tenderness of the left upper arm, which hindered arm elevation. A radiograph of the left humerus demonstrated an osteolytic lesion with a periosteal reaction involving the diaphyseal region (Figure 1). Laboratory studies revealed a red blood cell count of 4.15×10^12^/L, hemoglobin (Hb) of 11.4 g/dL, platelet count of 249×10^9^/L, white blood cell count of 26.9×10^9^/L, C-reactive protein level of 7.7 mg/dL (normal range: 0-0.2 mg/dL), and lactate dehydrogenase level of 344 IU/L (normal range: 97-247 IU/L). A peripheral blood smear demonstrated no blast cells, and blood cultures were all negative. Coronal T1-weighted magnetic resonance imaging (T1WI) of the left humerus demonstrated diffuse low signal intensity of the bone marrow (Figure 2A), whereas coronal short tau inversion recovery (STIR) imaging demonstrated this as an area of local high signal intensity in the diaphysis (Figure 2B). Subcutaneous tissues adjacent to the brachial muscles exhibited high signal intensity on the axial STIR image (Figure 2C). Computed tomography showed permeative destruction of the humeral bone. The diagnosis was acute osteomyelitis.

**Figure 3:**
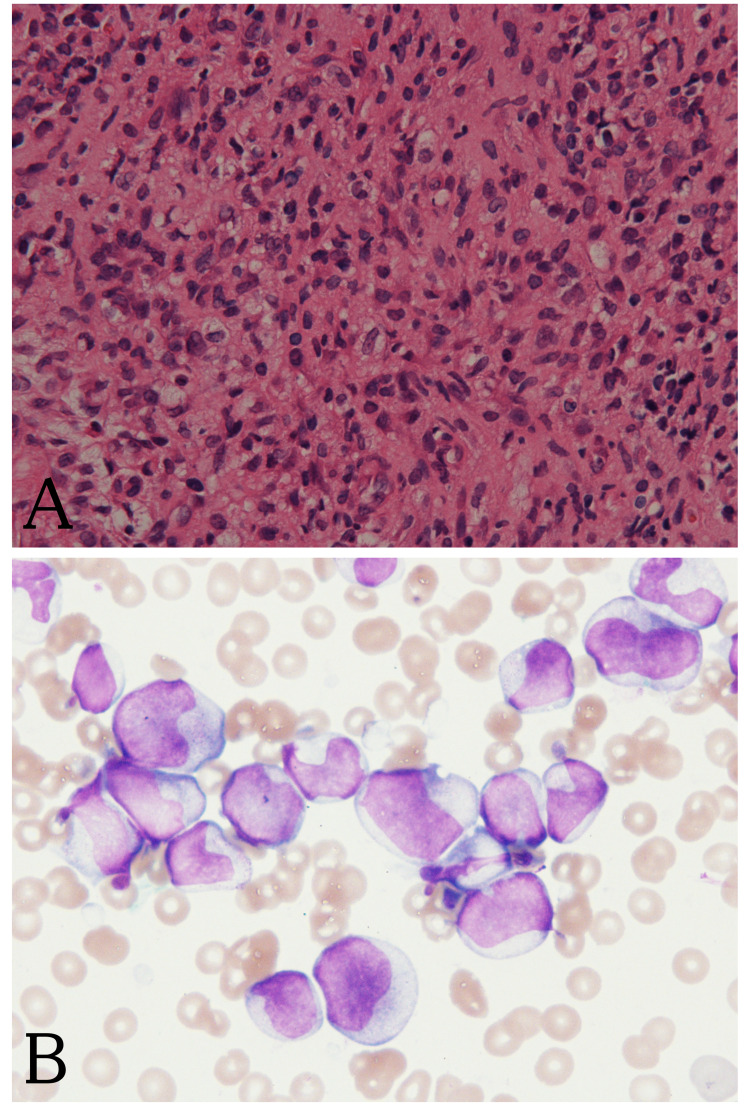
Pathological examination of the humeral lesion and iliac crest bone marrow. Section from the humeral lesion showing extramedullary monoblastic hyperplasia (×40 magnification, hematoxylin and eosin) (A). Iliac crest bone marrow biopsy showing blasts with cleaved and lobed nuclei, and azurophil granules (×1000 magnification, Wright-Giemsa stain) (B).

Despite treatment with not only wide-spectrum antibiotics including cefazolin, vancomycin, and rifampicin for 12 days but also immunoglobulin for the osteomyelitis, the fever persisted, and the osteomyelitis worsened. Curettage of the humeral lesion and subsequent histopathological analysis were performed in order to control the infection, obtain a definitive diagnosis, and devise suitable therapy. An initial review of the bone biopsy specimen was consistent with myeloid leukemia with monoblastic hyperplasia (Figure 3A). Immunostaining revealed atypical spindle-shaped cells that were positive for CD4, CD33, CD56, and lysozyme, being compatible with AML M5. An iliac crest bone marrow biopsy was then performed to confirm the diagnosis. The bone marrow was packed with monocytic blasts, and atypical monocytes accounted for 80% of the leukocytes (Figure 3B). These cells consisted mainly of monocytes with cleaved and lobed nuclei, and promonocytes with microscopic azurophil granules. The blasts were positive for myeloperoxidase, CD13, and CD33. On the basis of the morphology, differential count, and immunophenotype of the blasts revealed by bone marrow aspiration, a final diagnosis of AML M5b was made. Cytogenetic examination of the bone marrow specimen identified the abnormal karyotype 46, XY, t(10;11)(p11.2;q23), in the blasts. Molecular analysis revealed the absence of FLT3-internal tandem duplication (ITD).

Although osteomyelitis usually develops in the metaphyseal and not the diaphyseal region, we misdiagnosed the present case as acute osteomyelitis. On the other hand, bone tumors showing osteolysis, such as Ewing’s sarcoma and eosinophilic granuloma, need to be considered in the differential diagnosis. As the bone was negative for S-100 and CD1a, the possibility of Ewing’s sarcoma or eosinophilic granuloma was ruled out.

After the diagnosis of AML, the patient received induction chemotherapy with etoposide, cytarabine, and mitoxantrone. One month later, after the completion of the induction chemotherapy, relief from the bone pain was achieved, and further four cycles of chemotherapy with etoposide, cytarabine, mitoxantrone, and idarubicin were administered.

**Figure 4:**
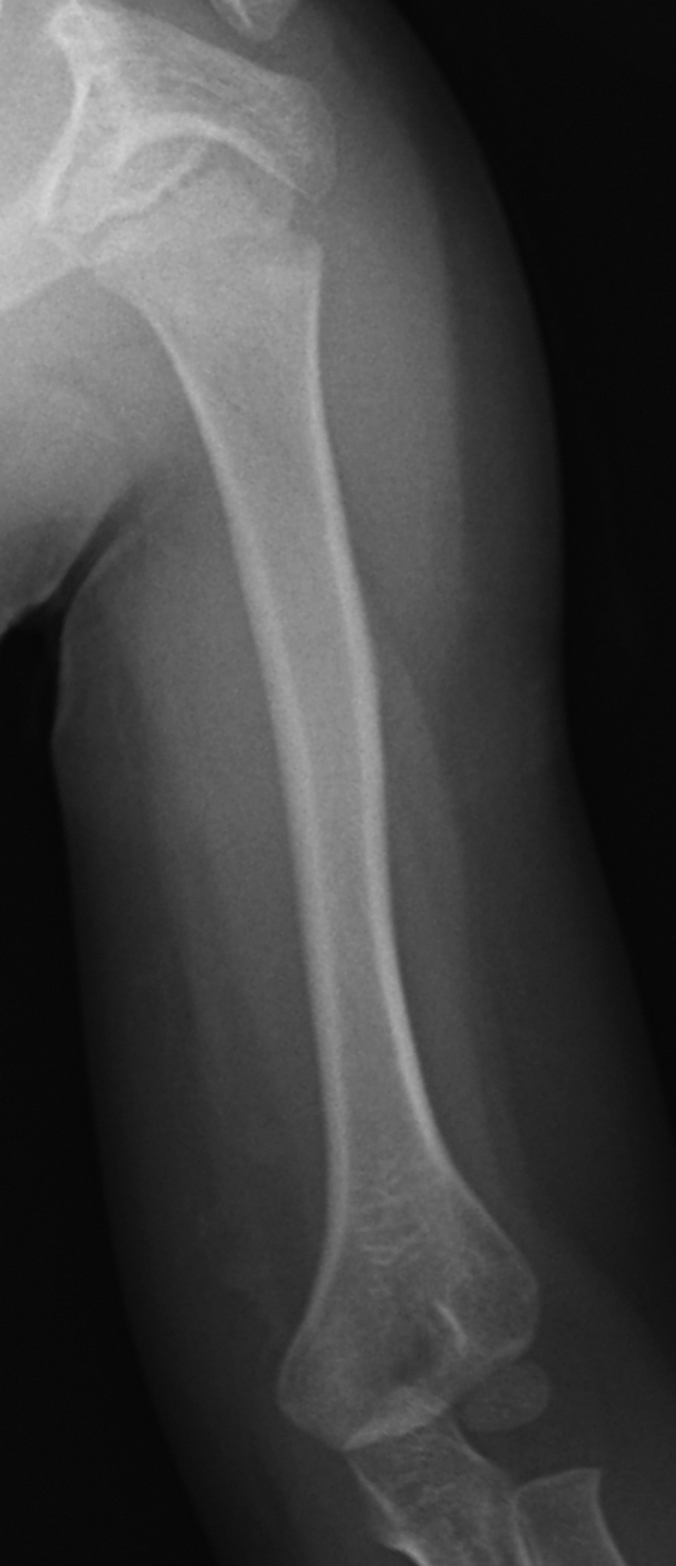
Anteroposterior radiograph of the left humerus taken 36 months after diagnosis showing complete resolution of the osteolytic bone lesion.

The patient is currently alive in remission, and radiography shows complete resolution of the osteolytic lesions after a post-diagnosis follow-up of 36 months (Figure 4).

## Discussion

This case highlighted two important clinical issues: skeletal pain can be the primary presenting symptom of AML in children, and MRI can provide evidence strongly suggestive of acute leukemia (AL), for which histopathological examination of the bone focus can yield diagnostically important information.

Children with AL may be referred to an orthopedist when the diagnosis is still unknown. Although it is well known among hematologists that AL is one of the most serious causes of skeletal pain, orthopedists may be unaware of this [2]. In fact, bone pain or radiographic abnormalities can be very frequent at presentation in cases of acute lymphoblastic leukemia (ALL) [3]. On the other hand, bone involvement on initial presentation is extremely rare in AML, and only six such cases have been reported in patients below the age of 15 years (Table 1) [4-8]. Bone pain in AL is caused by the proliferation of leukemic cells in the bone marrow and pressure effects within the medullary canal and under the periosteum [9]. Skeletal lesions are more frequent in children because the child’s small marrow reserve is replaced more quickly by leukemic cells [10]. Such bone manifestations are found more often in lymphoid leukemia than in myeloid leukemia [11].

Although features such as fever, pallor, bleeding tendency, and presence of leukemic cells may help confirm AL, an iliac crest bone marrow biopsy can allow cytological and cytochemical identification of blasts [12]. However, the diagnosis of AL may be especially difficult in children, as the clinical presentation may sometimes be only musculoskeletal pain or congenital infection, and any blood abnormalities may be nonspecific. Clinical decision-making based on an iliac crest bone marrow biopsy may not be easy if the bone lesion is present at a single site or if complete blood cell counts are normal. Among the six cases described previously, two had only one bone lesion, and no peripheral leukemic cells were evident at the first visit in three (Table 1). Furthermore, these cases were initially misdiagnosed as discitis or septic arthritis. Similar to the misdiagnosed cases, the initial diagnosis in the present case was acute osteomyelitis. It was difficult to consider the possibility of AL because not only was there only one bone lesion but also peripheral leukemic cells were absent. Only one of the six previously reported cases was investigated by bone biopsy (Table 1), and in the present case, histopathological examination of the bone focus yielded diagnostically useful data. This suggests that bone biopsy should be considered in cases of treatment-resistant osteolysis regardless of whether peripheral leukemic cells are absent.

**Table 1:** AML presenting with skeletal symptoms at initial presentation in previously reported pediatric cases. AML: acute myeloid leukemia; NA: not applicable.

Authors	Age and sex	Bony sites	Systemic symptoms	Appearance of blasts	First diagnosis	Peripheral bone marrow biopsy	Iliac crest bone marrow biopsy
Fisher et al. [4]	One year, female	Skull, radius, ulna, hand, femur, tibia	Fever, pallor, lymphadenopathy	First visit	AML	-	+
Franco et al. [5]	Eight months, male	Skull, spine, pelvis, femur	Fever	First visit	AML	-	+
Tsujioka et al. [6]	Three years, female	Skull, spine, pelvis, femur, tibia	Fever	First visit	AML	-	+
Chell et al. [7]	10 years, male	Spine	Fever	NA (not first visit)	Discitis	+	+
	Nine years, male	Knee	Bleeding	Two weeks after the first visit	Septic arthritis	-	+
Overholt et al. [8]	Two years, male	Pelvis, femur	Fever, lethargy	Four weeks after the first visit	Septic arthritis	-	+
Present case	One year, male	Humerus	Fever	Two weeks after the first visit	Acute osteomyelitis	+	+

Several previous reports have discussed the utility of MRI for distinguishing AL from osteomyelitis or bone tumor (Table 2) [13-20]. In children older than one year, upon comparison with surrounding muscular intensity, the marrow signal intensity on T1WI appears high in the humerus [13]. Therefore, in children aged above one year, diffuse low signal intensity on T1WI of the bone marrow in the humerus suggests a nonphysiological condition. The present case showed diffuse low signal intensity on T1WI and high signal intensity in the STIR image in the diaphyseal region of the humerus. The initially decreased signal intensity of the bone marrow reflected infiltration of leukemic cells. Previous reports have suggested that an abnormal MRI signal in AL is generally diffuse and homogeneous in the bone marrow [14]. However, the diffuse low signal intensity of the bone marrow on T1WI is not specific for AL [15]. These patients are often misdiagnosed as having an infectious disease or bone tumor. In osteomyelitis, although similar findings are evident in the bone marrow, the most common site of osteomyelitis is the metaphyseal region. Ill-defined margins with a wide transition zone between the normal and affected bone are recognized in all patients with acute osteomyelitis [16]. Intramedullary and extramedullary fat globules on T1WI are frequently observed in acute osteomyelitis [17]. The increasing intramedullary pressure leads to septic necrosis with the death of the lipocytes and the release of lipid. Furthermore, a recent report has proposed that the penumbra sign is specific for subacute osteomyelitis [18]. This is a higher signal intensity feature of the thin layer of granulation tissue that lines the abscess cavity on T1WI. It is also important to rule out the possibility of a bone tumor, especially Ewing’s sarcoma or eosinophilic granuloma. A sharp and defined margin, optimally visualized on T1WI, is the most significant feature of Ewing’s sarcoma [19]. Eosinophilic granuloma has been reported to show both a low signal endosteal rim and limited peritumoral bone marrow edema on STIR images [20]. It is necessary to be mindful of the T1WI features of the bone marrow in the region adjacent to the site of the localized musculoskeletal symptoms. If T1WI depicts diffuse low signal intensity of the bone marrow, this is an important clue to the possibility of AL in children more than one year of age. Although MRI is highly sensitive for detecting bone marrow infiltration, its specificity is poor, and bone biopsy remains the mainstay in the workup of these patients.

**Table 2:** Differential diagnosis with MRI findings of acute leukemia. MRI: magnetic resonance image; T1WI: T1-weighted magnetic resonance image; T2WI: T2-weighted magnetic resonance image; STIR: short tau inversion recovery.

	MRI	T1WI	T2WI	Pattern of enhancement	Common site
Acute leukemia	Diffuse and homogeneous in bone marrow on T1WI	Low signal intensity	High signal intensity	Increased	Diaphysis
Acute osteomyelitis	Intramedullary and/or extramedullary fat globules and ill-defined medullary, cortical, and soft tissue signal change	Low–intermediate signal intensity	High signal intensity	Increased rim enhancement surrounding a necrotic or devitalized tissue	Metaphysis
Subacute osteomyelitis	Penumbra sign on T1WI and a well-defined rim of low signal intensity surrounding the active disease	Low–intermediate signal intensity	High signal intensity	Granulation tissue lining a cavity enhanced strongly	Metaphysis, extending to the diaphysis or epiphysis of the long bone
Ewing’s sarcoma	A sharp and defined margin of the bone lesion on T1WI and skip lesions affecting the bone	Low signal intensity	High signal intensity	Contrast enhancing soft tissue mass derived from bone	Metadiaphysis and metaphysis
Eosinophilic granuloma	An endosteal rim of low signal intensity surrounding the main lesion on STIR image and limited peritumoral bone marrow edema on STIR image	Low–intermediate signal intensity	High signal intensity	Postcontrast enhancement with or without soft tissue mass	Diaphysis and metaphysis, extending to the physis and epiphysis of the long bone

We believe that in the currently improved clinical environment, reevaluation of the diagnostic procedure for patients with AML presenting as a skeletal disease is essential. In children with AL showing musculoskeletal involvement, diagnostic delay has been reported [21]. The progress made in the treatment of AL emphasizes the need for precise and speedy diagnosis, as early diagnosis significantly decreases morbidity and mortality [22]. Not only MRI findings but also bone biopsy would further contribute to this process. The presence of cytological features in the bone focus may prompt the specific use of iliac crest bone marrow biopsy for a disease that is otherwise difficult to diagnose early.

## Conclusions

A single skeletal osteolytic lesion can be the initial symptom in pediatric cases of AML. Clinicians should be aware of AML as a differential diagnosis when assessing children with single skeletal manifestations and associated systemic symptoms. MRI can detect leukemic bone marrow involvement as a diffuse low signal in T1WI before blast cells have appeared in the peripheral blood. Because MRI of the leukemic bone marrow is highly sensitive but not specific, a bone biopsy should be performed and is capable of providing a correct diagnosis.

## References

[1] Rogalsky RJ, Black GB, Reed MH (1986). Orthopaedic manifestations of leukemia in children. J Bone Joint Surg Am.

[2] Jones OY, Spencer CH, Bowyer SL, Dent PB, Gottlieb BS, Rabinovich CE (2006). A multicenter case-control study on predictive factors distinguishing childhood leukemia from juvenile rheumatoid arthritis. Pediatrics.

[3] Rajantie J, Jääskeläinen J, Perkkiö M, Siimes MA (1985). Prognostic significance of primary bone changes in children with acute lymphoblastic leukemia. Pediatr Radiol.

[4] Fisher D, Ruchlemer R, Hiller N, Blinder G, Abrahamov A (1997). Aggressive bone destruction in acute megakaryocytic leukemia: a rare presentation. Pediatr Radiol.

[5] Franco A, Lewis KN, Blackmon JM, Manaloor EJ (2010). Hyperostosis - an unusual radiographic presentation of myelodysplastic syndrome transformed to acute myeloid leukemia. J Radiol Case Rep.

[6] Tsujioka T, Sugiyama M, Ueki M (2018). Difficulty in the diagnosis of bone and joint pain associated with pediatric acute leukemia; comparison with juvenile idiopathic arthritis. Mod Rheumatol.

[7] Chell J, Fernandes JA, Bell MJ (2001). The orthopaedic presentation of acute leukaemia in childhood. Ann R Coll Surg Engl.

[8] Overholt K, Guinipero TL, Heerema NA, Loken MR, Kahwash SB (2015). Promyelocytic leukemia with no retinoic acid receptor alpha abnormality but with RUNX1T1 insertion to chromosome 7q: a classification and management dilemma. Case Rep Hematol.

[9] Barbosa CM, Nakamura C, Terreri MT, Lee ML, Petrilli AS, Hilário MO (2002). [Musculoskeletal manifestations as the onset of acute leukemias in childhood]. J Pediatr (Rio J).

[10] Gallagher DJ, Phillips DJ, Heinrich SD (1996). Orthopedic manifestations of acute pediatric leukemia. Orthop Clin North Am.

[11] Robazzi TC, Barreto JH, Silva LR, Santiago MB, Mendonça N (2007). Osteoarticular manifestations as initial presentation of acute leukemias in children and adolescents in Bahia, Brazil. J Pediatr Hematol Oncol.

[12] Moody A, Simpson E, Shaw D (1989). Florid radiological appearance of megakaryoblastic leukaemia--an aid to earlier diagnosis. Pediatr Radiol.

[13] Yoshikawa T, Tanizawa A, Suzuki K (2016). The usefulness of T1-weighted magnetic resonance images for diagnosis of acute leukemia manifesting musculoskeletal symptoms prior to appearance of peripheral blood abnormalities. Case Rep Pediatr.

[14] Kato M, Koh K, Kikuchi A (2011). Case series of pediatric acute leukemia without a peripheral blood abnormality, detected by magnetic resonance imaging. Int J Hematol.

[15] Silva JR Jr, Hayashi D, Yonenaga T (2013). MRI of bone marrow abnormalities in hematological malignancies. Diagn Interv Radiol.

[16] Shimose S, Sugita T, Kubo T, Matsuo T, Nobuto H, Ochi M (2008). Differential diagnosis between osteomyelitis and bone tumors. Acta Radiol.

[17] Davies AM, Hughes DE, Grimer RJ (2005). Intramedullary and extramedullary fat globules on magnetic resonance imaging as a diagnostic sign for osteomyelitis. Eur Radiol.

[18] McGuinness B, Wilson N, Doyle AJ (2007). The "penumbra sign" on T1-weighted MRI for differentiating musculoskeletal infection from tumour. Skeletal Radiol.

[19] Henninger B, Glodny B, Rudisch A (2013). Ewing sarcoma versus osteomyelitis: differential diagnosis with magnetic resonance imaging. Skeletal Radiol.

[20] Davies AM, Pikoulas C, Griffith J (1994). MRI of eosinophilic granuloma. Eur J Radiol.

[21] Brix N, Rosthøj S, Herlin T, Hasle H (2015). Arthritis as presenting manifestation of acute lymphoblastic leukaemia in children. Arch Dis Child.

[22] Sinigaglia R, Gigante C, Bisinella G, Varotto S, Zanesco L, Turra S (2008). Musculoskeletal manifestations in pediatric acute leukemia. J Pediatr Orthop.

